# The Value of a Name in Dentistry

**Published:** 1887-05

**Authors:** 


THE VALUE OF A NAME IN DENTISTRY.—The name of the American dentist, Thomas Evans, who has resided in Paris many years, is well known throughout Europe and the United States. Under the empire he was the court dentist, the Emperor and the beautiful Eugenio, and perhaps also the Prince Imperial, submitted to him the care of their teeth, and when, after the disaster of Sedan the third republic was proclaimed and Eugenie withdrew precipitately from Paris, it was the dentist Thomas Evans at whose house she took refuge, and who escorted her to England. Many of the royal and princely families of Europe patronized Thomas Evans, and as matter of course fashionable people and wealthy Americans abroad followed their example. The name of Evans became famous, and the dentist Evans became very rich. Being overburdened with business, he took into his office his nephew, John Evans, not as a partner but as an assistant, making many promises as to what he would do for him. But the nephew, John Evans, who was himself a skillful practitioner, becoming finally weary of promises, left his uncle and set up for himself. From that time there was a bitter war between the two. The elder Evans determined that no rival Evans, even though he were the son of his brother, should attract the favor of the public and share his fame. This happened before the fall of the empire. The younger Evans was the son of Rodolphe Henri Evans and Elizabeth d'Oyley. The mother being of noble birth, John Evans on his marriage sought and obtained in 1879 from the Court of Common Pleas of Philadelphia authority to change his name to John d'Oyley, and on his return to France assumed the title of Marquis d'Oyley, as it appears he was warranted in doing in right of his mother. It was on this change of name the elder Evans fought his battle. He sought at first the intervention of the Emperor to compel John Evans to abandon the name of Evans in his practice as a dentist, and compel him to use the name of d'Oyley, by which he was known in society. As the Emperor declined to interfere, the elder Evans took the case into the courts. The fight thus commenced has been going on, according to a Paris chronicle, for twenty years—"twenty years of intestine struggles, of vexations and gnashing of teeth." The first suit the elder Evans lost. The second, to interdict his nephew, John Evans, from bearing the name of Evans and constrain him to call himself Marquis d'Oyley, the uncle gained. John Evans then appealed to the Third Chamber of the Court of Paris. He only desired, he said, to have the right to call himself John Evans d'Oyley. The court appears to have appreciated his modesty. It gave him more than he asked. It decided that, in the practice of his profession, he had the right to use the name by which he was baptized, and that of Marquis d'Oyley in society and among his friends. The elder Evans was condemned to pay the costs of the suits he had instituted. So ends at last a quarrel that has long been the talk of Paris.—*Sun.*

